# The impacts of caring for children with inherited metabolic diseases for families: a cross-sectional study

**DOI:** 10.1186/s13023-026-04434-y

**Published:** 2026-06-10

**Authors:** Andrea J. Chow, Isabel Jordan, Nicole Pallone, Maureen Smith, Pranesh Chakraborty, Jamie Brehaut, Alicia K. J. Chan, Eyal Cohen, Sarah Dyack, Ian D. Graham, Cheryl R. Greenberg, Robin Hayeems, Michal Inbar-Feigenberg, Shailly Jain-Ghai, Sara Khangura, Jennifer J. MacKenzie, Nathalie Major, John J. Mitchell, Stuart G. Nicholls, Amy Pender, Murray Potter, Chitra Prasad, Andreas Schulze, Komudi Siriwardena, Kathy N. Speechley, Sylvia Stockler, Monica Taljaard, Yannis Trakadis, Jagdeep Walia, Kumanan Wilson, Beth K. Potter

**Affiliations:** 1 School of Epidemiology and Public Health, University of Ottawa, 600 Peter Morand Crescent, Office 207C, Ottawa, ON K1G 5Z3 Canada; 2 Patient partner, Squamish, Canada; 3 Department of Pediatrics, Children’s Hospital of Eastern Ontario, Ottawa, Canada; 4 Patient Partner, Ottawa, Canada; 5 Patient Partner, Sparwood, Canada; 6 Division of Clinical & Metabolic Genetics, The Hospital for Sick Children, Toronto, Canada; 7 Department of Pediatrics and Child Health, University of Manitoba, Winnipeg, Canada; 8 Methodological and Implementation Research, Ottawa Hospital Research Institute, Ottawa, Canada; 9 Department of Medical Genetics, University of Alberta, Edmonton, Canada; 10 Department of Pediatrics, University of Toronto, Hospital for Sick Children, Toronto, Canada; 11 Department of Pediatrics, Dalhousie University, Halifax, Canada; 12 Child Health Evaluative Sciences, University of Toronto, Hospital for Sick Children, Toronto, Canada; 13 Department of Medicine, Queen’s University, Kingston, Canada; 14 Montreal Children’s Hospital, Montreal, Canada; 15 Office for Patient Engagement in Research Activities, Ottawa Hospital Research Institute, Ottawa, Canada; 16 Genetics & Metabolics Clinic, McMaster Children’s Hospital, Hamilton, Canada; 17 Department of Pathology and Molecular Medicine, McMaster University, Hamilton, Canada; 18 Department of Pediatrics, Western University, London, Canada; 19 Department of Biochemistry, University of Toronto, Toronto, Canada; 20 BC Children’s Hospital, Vancouver, Canada; 21 Medical Genetics, Montreal Children’s Hospital, Montreal, Canada; 22 Department of Pediatrics, Queen’s University, Kingston, Canada; 23 Department of Medicine, University of Ottawa, Ottawa, Canada; 24 Bruyère Health Research Institute, Ottawa, Canada

**Keywords:** Family-centred care, Pediatrics, Healthcare management, Inherited metabolic diseases, Rare diseases

## Abstract

**Background:**

Children with inherited metabolic diseases (IMDs) often have high care needs that require extensive involvement of family caregivers. This study aimed to describe caregiver experiences, including management of children’s needs at home, and the impacts of that management on family time, finances, and caregiver and child health-related quality of life (HRQoL).

**Methods:**

In this sub-study within a larger cohort study, participants were family caregivers of children ≤ 12 years old diagnosed with an IMD, from across Canada. We collected cross-sectional data using an online questionnaire consisting of validated measures and team-developed questions, and analyzed data descriptively.

**Results:**

Seventy-one caregivers participated and reported using a range of supports and interventions to care for their children with IMDs, with dietary supports being most common (77.1%). Twenty-seven participants (45%) reported spending ≥ 4 extra hours per week assisting their child with care supports at home and 28 reported ≥ 6 missed days of work by caregivers in the past year for IMD care. All participants reported fulfillment from caregiving tasks, although 82% reported mental struggles related to caregiving. Based on caregiver reports, children with IMDs had lower HRQoL relative to population normative data. We observed lower median scores for several HRQoL subscales among children with IMDs classified as progressive with multi-system involvement relative to other IMDs.

**Conclusion:**

We identified important impacts of IMD care for family caregivers. Relief from high levels of caregiving responsibility and time should be a priority for the development of healthcare policies and interventions, to improve health and well-being for both children and their caregivers.

**Supplementary Information:**

The online version contains supplementary material available at 10.1186/s13023-026-04434-y.

## Background

Inherited metabolic diseases (IMDs) are a heterogenous group of more than 1,400 individually rare genetic conditions, often diagnosed in early childhood, that have a collective estimated global prevalence of 50.9 in 100,000 live births [1, 2]. Some IMDs present a risk of acute metabolic decompensation, where the build-up of toxic metabolites can be life threatening, and some involve multiple organ systems and may be progressive even with available treatment. The physical and psychological well-being of children, often examined in research as child health-related quality of life (HRQoL), is an important outcome to family caregivers [3] but previous research is inconsistent about the impact of IMDs on child HRQoL. Several studies have found that family caregivers of children with IMDs, or children themselves, perceive children with IMDs to have lower HRQoL compared to the general pediatric population [4–10], though other studies have found no or few significant differences [11–17]. 

Higher caregiving impacts and greater disease severity may be associated with lower child HRQoL for children with IMDs [18]. Many children with IMDs have complex and intensive health care needs which require dietary management and regular monitoring and treatment, responsibilities that often fall to family caregivers on a day-to-day basis, especially for young children [19–21]. Parents whose children have chronic conditions, particularly conditions that are medically complex and characterized by high health system utilization and service needs [22], report experiencing chronic parenting stress and worse mental health than parents of children without chronic disease [23–29]. In quantitative studies, caregivers of children with IMDs frequently reported burnout, stress, and reduced quality of life due to the intensity of their care responsibilities [30–38]. Authors have reported that caregivers require additional time beyond typical childcare to manage aspects of IMD care [39–42], sometimes quitting jobs or reducing work hours to do so [32, 36, 42–45]. Family caregivers have described stress and anxiety about children’s health status and prognosis, child healthcare experiences, and impacts on employment opportunities in several qualitative studies [46–51]. In addition to its importance as a mental health outcome, chronic stress is also a risk factor for long-term adverse physical health outcomes, including: cardiovascular disease, type 2 diabetes and immune dysfunction [52–54]. 

Many studies of the impact of IMDs focus on one or a few IMDs, or on a specific aspect or outcome of caregiving for pediatric IMDs, making it challenging to identify outcomes common across IMDs. As there are common care elements across a broad range of IMDs as well as ones specific to groups of IMDs with similar care requirements, understanding the common challenges to IMD management, as well as variations across groups of IMDs, can help in measuring the overall impact of IMDs. Understanding these challenges is essential to guide the development of strategies that can be implemented widely to support families and has the potential to support the integration of family impacts into the consideration of the value of disease-modifying treatments that improve outcomes for IMDs.

We describe a cross-sectional sub-study embedded within a larger prospective cohort study about family-centred care for children with IMDs [55]. Our sub-study’s aim was to describe caregiver experiences with caring for children with IMDs, including management of children’s needs at home, and the impacts of that management on family time, finances, and quality of life. Our specific objectives were to:

1. Describe caregiver experiences using care supports and interventions to assist in (a) managing their child’s IMD care and (b) supporting family members;
2. Describe the impact of pediatric IMDs on caregiver employment, household finances, and quality of life (QoL); and,
3. Describe HRQoL in children with IMDs, using caregiver reports, and describe how HRQoL varies by child age and typical IMD trajectory.

## Methods

### Study design

This sub-study reports on data collected in the baseline questionnaire of a cohort study that aimed to comprehensively understand the healthcare experiences of children with IMDs and their families, as noted in the published study protocol [55]. We report this cross-sectional sub-study in accordance with the Strengthening the Reporting of Observational Studies in Epidemiology (STROBE) guidelines (see supplementary material, checklist) [56]. We co-designed the larger cohort study with three patient/family partner co-investigators on the research team (IJ, NP, MS) who are caregivers of children with rare disease and/or individuals with rare disease. An additional group of 11 family and patient advisors provided feedback on the questionnaire content during its development.

### Study participants

Participants in the cohort study were family caregivers of children diagnosed with an IMD. For each participating family, one caregiver was identified by the family as the “designated caregiver” to provide data regarding one child in their family with an IMD (“designated child”). Eligibility criteria for the cohort study were that the caregiver: was a Canadian resident; had a self-assessed ability to complete study questionnaires in English; and had a child who was 12 years or younger at recruitment with an IMD diagnosis, and who was receiving health care from one of 11 participating Canadian metabolic centres located in academic pediatric hospitals. Our focus was on caregiving, so child age was restricted to ≤ 12 years as adolescents with chronic conditions typically have different health care needs than younger children, and potentially different priorities from, and interactions with, caregivers in managing their care [57, 58]. With respect to IMD diagnoses, clinician members of the study team categorized IMDs into one of three broad clinical course trajectories, with different implications for health needs and healthcare use: (a) chronic and generally non-progressive; (b) acute episodes of severe illness with or without accompanying chronic multi-system sequelae; and (c) multi-system and progressive even with available treatment. Diagnoses of 30 IMDs included in a previous Canadian pediatric cohort study [59, 60] were eligible for this study (Additional File 1). To increase representation of trajectory (c), children were also deemed to be eligible if they had an IMD that involved at least three organ systems and manifested chronic, progressive complications, even with available treatment. Eligibility for each potential participant was evaluated by clinician investigators at participating metabolic centres. Participants included in this sub-study also had to have contributed data, any amount, to the cohort study’s baseline questionnaire.

Potential participants were informed of the cohort study by clinical team members at participating centres, in person or by telephone or email. Interested caregivers provided an email address at an online link to receive more information about the study and complete a web-based screening questionnaire. From those who completed the screening questionnaire, we used a purposive, maximum variation sampling approach [61, 62] to form a heterogenous sample with diversity on characteristics anticipated to be associated with children’s healthcare needs and experiences: metabolic clinic from which children were recruited, child’s age (years, categorized 0–3, 4–6, 7–9, > 9), IMD, IMD clinical course trajectory, travel time from home to study centre (≤1 h, > 1 – ≤4 h, > 4 h), and child’s sex assigned at birth. In the larger cohort study, in order to collect sufficient data for analysis, we prioritized the selection of participants who expected the designated child to have multiple health care encounters per month during the study. After finalizing the protocol but prior to selecting participants from the screening questionnaire results, we decided to exclude participants who expected to have no encounters over the cohort study period and applied this rule consistently during participant selection. When a person completed the screening questionnaire, we assessed whether their characteristics of interest would contribute to the diversity of the sample and decided whether to invite them. Participants provided informed consent and were enrolled in the cohort study on a rolling basis between November 2020 and March 2022.

### Data collection

Participants completed the cohort study’s web-based baseline questionnaire between December 2020 and May 2022. Study data were collected and managed using Research Electronic Data Capture (REDCap) hosted at the Children’s Hospital of Eastern Ontario [63, 64]. Participants received up to two reminders to complete the questionnaire.

### Variables and measures

The questionnaire was pilot tested with 11 family/patient advisors prior to study commencement. We collected data on caregiver, child and household characteristics, including caregiver’s highest education level attained and employment status, child age and IMD, and gross household income in the past year. Due to the potentially identifying nature of IMD diagnoses, we did not report on specific IMD diagnoses; however, we grouped participants by typical clinical course trajectory (chronic and non-progressive, acute and episodic, and multi-system and progressive). In addition, we grouped IMD diagnoses using the first tier of classification (e.g., disorder of energy metabolism) in the Orphanet classification of rare inborn errors of metabolism [65]. 

Data on supports needed and accessed for the child and family (objective 1) were collected using team-developed, closed-ended questions (Additional File 2). We asked caregivers to report on the treatments, therapies, services, products, or equipment (hereafter, “care supports and interventions”) used to manage the child’s IMD, including: types of care supports and interventions, perceived difficulty of, and time spent per week, managing care supports and interventions at home, and adequacy of care supports and interventions received over the past 12 months. We also sought caregivers’ perspectives on supports for the family: types of supports received, perceived degree of difficulty accessing supports, adequacy of the amount of any supports received, and supports that were unavailable when needed.

To pursue objective 2, we used team-developed closed-ended questions to collect information from caregivers about the employment and financial impacts of child IMD management (e.g., missed days of work, whether a caregiver ever had to leave a job or reduce work hours, out-of-pocket expenses; Additional File 2).

Caregiving-related QoL was assessed using the CarerQoL instrument, which has been used in studies of parents of children with chronic conditions [66–68], including rare diseases [69–71]. It has two components: the CarerQol-7D, seven items which each assess a dimension of caregiver QoL (relational problems, mental health problems, problems combining daily activities with care-tasks, financial problems, physical health problems, fulfilment from caregiving, and support), and the CarerQol-VAS, a visual analog scale which measures subjective well-being on a scale of 0–10, with a higher score indicating more positive well-being. We reformatted the measure for use in REDCap, incorporating check boxes for the 7D and a scale of 0-100 for the VAS.

We measured child HRQoL using the Child Health Questionnaire – Parent Form 50 [72] (CHQ-PF50) for children ≥ 5 years or the Infant and Toddler Quality of Life Questionnaire – 47-item short-form (ITQoL-SF47) [73] for children < 5 years. Both are parent-reported measures with established psychometric properties [73–75]. The CHQ-PF50 is composed of 50 items, which are summed to create scores on 12 subscales and aggregated to form two summary measures [72]. The ITQoL-SF47 is composed of 47 items which are summed to create scores on ten subscales [76]. Items on both the CHQ-PF50 and ITQoL-SF47 are summed and transformed into scores of 0–100, with higher scores indicating more positive well-being on a subscale or summary measure [72, 76]. The CHQ-PF50 and the ITQoL-SF47 each contain two subscales related to ‘parental impact – emotional’ and ‘parental impact – time’. We report the results for these subscales with the caregiving-related QoL results.

### Analyses

We primarily analyzed data descriptively, reporting continuous variables using means and standard deviations or medians and the first and third quartiles (Q1, Q3), and categorical variables using counts and proportions, as appropriate. We decided post-hoc to analyze and report the statistical significance of the associations between typical IMD trajectory and employment and financial impacts, child HRQoL, and caregiver QoL, interpreted using *p* < 0.05 as the threshold for statistical significance, and we reported 95% confidence intervals for IMD care support types stratified by child age and IMD trajectory (described in relevant sections below). We also decided post-hoc to explore associations between child sex and child HRQoL. Item missing data were less than 12% for all analyses and less than 5% for most analyses, and we handled them using casewise deletion for each analysis. We performed all statistical analyses using SAS version 9.4 (SAS Institute, Inc, Cary, NC).

#### Supports/interventions for the IMD and for the family

We classified supports and interventions used to manage a child’s IMD as: diet-related; pharmaceutical and physical; learning and developmental; devices and equipment; or other. We report counts and proportions for these supports. We collected time spent on IMD-related supports categorically (e.g.,<1 h, 1 to < 4 h, ≥ 4 h per week). To describe the minimum time spent by families on IMD-related tasks, we created new minimum time variables for each task and assigned the lowest possible value based on the category selected (e.g., for the category ‘1 to < 4 hours’, minimum time = 1 h). We summed all minimum time variables to create a variable for the overall minimum time for IMD caregiving tasks. We also report counts and proportions about supports for families.

In addition to the descriptive analyses, we analyzed the use of IMD care support and intervention types stratified by the IMD clinical trajectory and child age (< 5 and ≥ 5 years), both of which we anticipated could have an impact on IMD care management, and present proportions and 95% confidence intervals. Child age and IMD trajectory were highly correlated in our cohort (Supplementary Table 1, Additional File 3), which made it necessary to conduct stratified analyses addressing both variables at the same time, using six categories (three IMD trajectories within each of two child age groups).

#### Impact of IMDs for caregivers: employment, household finances, and caregiving-related QoL

We report counts and proportions for caregiver employment, impacts related to household finances, and each dimension of the CarerQoL; and we report the median score for well-being (based on the VAS) from the CarerQoL, re-proportioned from a scale of 0-100 to 0–10. We stratified these variables by IMD clinical trajectory and child age and evaluated differences among groups, using Fisher’s exact test to compare proportions and the Kruskal-Wallis *H* test to compare median scores.

#### Health-related quality of life in children with IMDs

We report medians with Q1-Q3 for child HRQoL subscale scores, within age groups (< 5 years, ≥ 5 years), overall and stratified by IMD trajectory. We made comparisons to normative data for general pediatric populations based on the effect size Hedges’ *g*, using Cohen’s guidelines to interpret the effect sizes as ‘small’ (0.2–0.5), ‘medium’ (0.5–0.8) or ‘large’ (> 0.8) [77]. We compared median child HRQoL scores across IMD clinical trajectories within age groups using the Kruskal-Wallis *H* test, and between child sex assigned at birth within age groups using the Wilcoxon rank-sum test.

## Results

### Participant, child and household characteristics

Of the 104 people who completed the screening questionnaire, all but one person were deemed eligible (99%) and 91 (88%) were invited to enroll in the cohort study. With consideration of the study’s maximum variation sampling approach, the remaining 12 eligible people were not invited to the study as their characteristics of interest were deemed too similar to the characteristics of the existing sample at the time of deciding whether to invite a caregiver. Of those invited, 82 (90%) consented to take part in the cohort study and 71 (78%) contributed data to this sub-study.

Most participants were mothers or stepmothers to the designated child (*n* = 64, 90%), college or university graduates (*n* = 57, 80%), and currently working full- or part-time (*n* = 50, 70%) (Table 1). 57% of designated children (*n* = 41) were aged 0–5 years. Twenty-nine children (41%) had an IMD whose trajectory was characterized as “acute and episodic”, 23 (32%) had a “chronic and non-progressive” IMD, and 19 (27%) had a “multi-system and progressive” IMD. Thirty-two (45%) designated children lived in households > 1 h away from the metabolic clinic. In 8% of households, a primary caregiver had immigrated to Canada within the previous ten years. In 52 (73%) households, there was at least one other child under the age of 18 years; in seven of these households, an additional child had an IMD diagnosis. Twenty-four (38%) households had an income of more than $120,000 in the past year.

**Table 1:** Participant, child, and household characteristics (*N* = 71)

	*n* (valid %)
**Participant characteristics**	
Relation to child	
Mother or stepmother	64 (90)
Father	7 (10)
Highest education level attained	
Secondary school or GED	14 (20)
College or vocational school graduate	17 (24)
Undergraduate degree	31 (44)
Graduate or post-graduate degree	9 (13)
Currently working full-/part-time for pay, yes	50 (70)
**Child characteristics**	
Age, years	
0–1	18 (25)
2–3	10 (14)
4–5	13 (18)
6–7	10 (14)
8–9	13 (18)
10–11	5 (7)
12–13	2 (3)
Sex assigned at birth, female^a^	40 (56)
IMD clinical trajectory	
Acute and episodic	29 (41)
Chronic and non-progressive	23 (32)
Progressive and multi-system	19 (27)
IMD classification	
Amino acid & other organic acid metabolism disorders	27 (38)
Energy metabolism disorders	24 (34)
Lysosomal diseases	13 (18)
Other	7 (10)
Has other chronic condition(s) besides IMD, yes	16 (24)
Metabolic clinic (by city location)	
Halifax	12 (17)
Hamilton	10 (14)
Ottawa	9 (13)
London	8 (11)
Kingston	8 (11)
Calgary	7 (10)
Toronto	6 (8)
Edmonton	5 (7)
Other^b^	6 (8)
**Household characteristics**	
Travel time to metabolic clinic	
1 h or less	39 (55)
More than 1 h and up to 3 h	17 (24)
More than 3 h	15 (21)
Number of primary caregivers	
1	5 (7)
2	57 (80)
3 or more	9 (13)
Any primary caregiver is a recent immigrant^c^, yes	6 (8)
Other children < 18 years living in the household	
None	19 (27)
1	31 (44)
2 or more	21 (30)
Other child(ren) in household with IMD diagnosis, yes (*N* = 52)	7 (13)
Household income, gross, past year (Canadian $)	
$60,000 or less	14 (22)
>$60,000 - $80,000	6 (10)
>$80,000 - $100,000	11 (17)
>$100,000 - $120,000	8 (13)
>$120,000	24 (38)
*Missing*	*8*

^a^ Sex of all other children reported as ‘male’

^b^ Montreal, Vancouver, Winnipeg

^c^ Immigrated to Canada 10 years or fewer prior to questionnaire completion

### Caregiver experiences using care supports and interventions to assist in managing their child’s IMD care and supporting family members (objective 1)

Sixty-five participants (93%) indicated the use of at least one type of care support/intervention by the family for managing their child’s IMD (Table 2). A majority of families used diet-related supports over the past 12 months (*n* = 54/71, 77%), most often including dietary recommendations from a healthcare professional (64%) and medical foods/formulas. A majority of families also used pharmaceutical and physical supports/interventions (59%), for example, prescription medications (56%) and exercise or physical support services (23%). Thirty participants (43%) reported that their families used learning and development supports/interventions, such as school assistance/accommodations.

**Table 2:** Care support/intervention usage and access over previous 12 months (*N* = 71)

Care support/intervention type	Used *n* (%) *N* = 71	Did not receive adequate amount *n*/*N* (%)
No care support/intervention types	5 (7)	--
At least 1 support/intervention type	65 (93)	--
* Missing*	*1*	--
*Diet-related supports/interventions*	54 (77)	--
Dietary recommendations from metabolic doctor / dietitian	45 (64)	1/45 (2)
Medical foods or formulas	33 (47)	2/33 (6)
Dietary supplements (not food/formula)	30 (43)	2/30 (7)
Dietary support services	6 (9)	1/6 (17)
*Pharmaceutical and physical supports/interventions*	41 (59)	--
Prescription drugs or medication	39 (56)	0/39 (0)
Exercise or physical support services	16 (23)	8/16 (50)
Other therapies^a^	5 (7)	1/5 (20)
*Learning and development supports/interventions*	30 (43)	--
School assistance or accommodation	22 (31)	7/22 (32)
Neuropsychological or cognitive assessments	12 (17)	1/12 (8)
Other supports^b^	6 (9)	2/6 (33)
*Devices and equipment*	16 (23)	--
Feeding tube	10 (14)	0/10 (0)
Assisted mobility aid (including wheelchair, other- adapted stroller, other-modified bike)	7 (10)	1/7 (14)
Other^c^	14 (20)	0/14 (0)
*Other care supports/interventions*	17 (24)	--
Home care	11 (16)	2/11 (18)
Respite care	9 (13)	6/9 (67)
Social services	8 (11)	2/8 (25)
Other ^d^	4 (6)	0/4 (0)

^a^Supplemental oxygen therapy, equine therapy, exercise prescription

^b^Early childhood intervention, speech therapy, behaviour support

^c^Home modifications, ventilator, CPAP machine, corrective lenses, hearing aid, and other – specified: Hoyer lift, central line, assistive communication devices, suction, ketones reader, AFOs, nebulizer, oximeter, handrail for tub, safety lancets for at-home blood collection, wrist braces, hospital bed and air mattress

^d^Transport services, and other – specified: inpatient admission, ophthalmologist, physiotherapy referral to train nurses

Regarding access to care supports/interventions, participants whose families used care supports/interventions over the previous 12 months generally felt that they received enough of those supports/interventions (Table 2). However, > 30% of participants whose families used respite care (*n* = 7/9, 78%), exercise or physical support services (*n* = 8/16, 50%), and school assistance or accommodation (*n* = 7/22, 32%) reported that they did not receive sufficient amounts of these supports/interventions. Furthermore, twenty participants (30%) reported that their families could not acquire at least one IMD care support/intervention type when needed over the previous 12 months, and ten of those participants reported not being able to acquire multiple supports (Supplementary Table 2, Additional File 3). The care supports/interventions most frequently unavailable when needed were school assistance or accommodation (*n* = 9, 14%) and medical foods or formulas (*n* = 5, 8%). When care supports/interventions were not available, the most commonly perceived reasons for unavailable care supports/interventions were: there was a waiting list, it was not available in the home province/territory, and the COVID-19 pandemic.

**Fig. 1:**
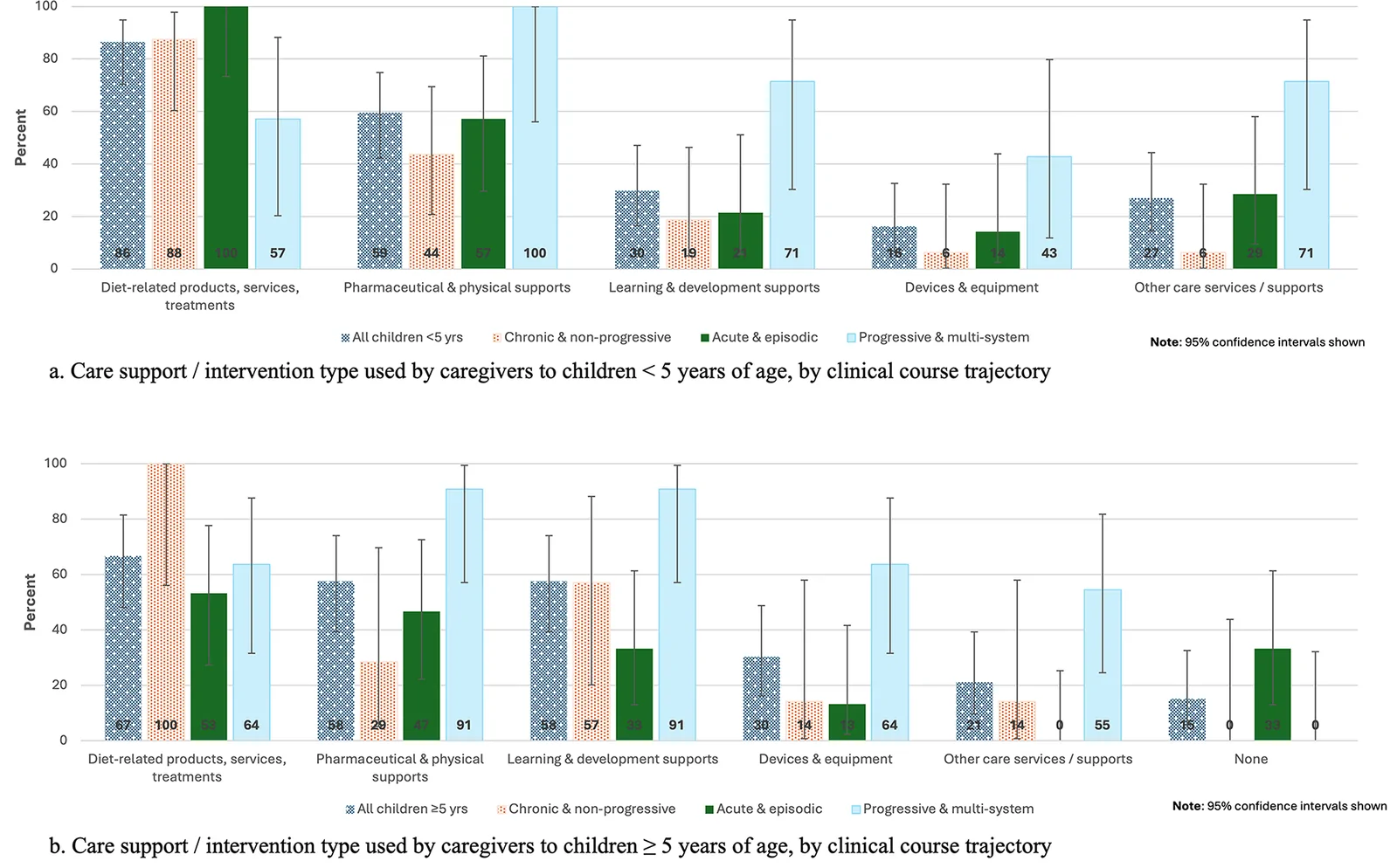
**A** Percents of children < 5 years old who used care supports over previous 12 months, by inherited metabolic disease clinical trajectory, with confidence intervals. **B.** Percents of children ≥ 5 years old who used care supports over previous 12 months, by inherited metabolic disease clinical trajectory, with confidence intervals

We observed some differences in use of care supports and interventions across IMD trajectories (Fig. 1a, children < 5 years; Fig. 1b, children ≥ 5 years). Notably, a higher proportion of caregivers to children with multi-system and progressive IMDs, relative to caregivers to children with chronic and non-progressive IMDs or acute and episodic IMDs, respectively, reported that their families used pharmaceutical and physical supports/interventions, learning and development supports/interventions, devices and equipment, and other care services/supports among children both < 5 and ≥ 5 years (Fig. 1a and b). In contrast, nearly all families with children with chronic and non-progressive IMDs and children < 5 years with an acute and episodic IMD used diet-related care supports/interventions, while approximately 60% of families with children with multi-system and progressive conditions did.

Forty (63%) participants perceived at least one care support/intervention type used at home to be very or somewhat difficult to manage (Table 3). Twenty-seven participants (45%) reported that their families spent a minimum of 4 h per week managing care supports/interventions at home, with one-third of those participants (*n* = 9) reporting their families spent at least 8 h per week. Of the families who used diet-related care supports/interventions, 11 (22%) spent > 4 extra hours per week, on average, planning and preparing meals due to their child’s IMD.

**Table 3:** Management of care supports at home (*N* = 71)

	*n* (%)
Difficulty managing care support types at home	
Somewhat or very difficult managing ≥ 1 care support type	40 (63)
No care support types used at home were difficult to manage	23 (36)
* Not applicable* ^*a*^	*6*
* Missing*	*2*
Minimum time spent per week assisting child with care supports at home^a^	
≥ 8 h	9 (15)
≥ 4 – <8 h	18 (30)
1 – <4 h	11 (18)
None	22 (37)
* Not applicable*	*6*
* Missing*	*5*
Extra time spent per week planning and preparing meals	
> 4 h	11 (22)
> 0–4 h	22 (44)
None	17 (34)
* Not applicable (did not use diet-related supports)* ^*b*^	*17*
* Missing*	*4*

^a^We did not ask about the degree of difficulty of, or the amount of time spent, managing the following care supports at home, as these support types were typically not accessed at home and/or not self-managed: School assistance or accommodation, neuropsychological or cognitive assessments, early childhood intervention, home care, respite care, social services, transport services

^b^Only asked if participants indicated use of dietary recommendations from metabolic doctor or dietitian, medical foods or formulations, or dietary supplements

Approximately half (*n* = 36, 51%) of participants reported that their families used at least one type of family support in the previous 12 months (Table 4). Nearly half of caregivers who reported using a family support indicated that they experienced difficulty acquiring at least one support (*n* = 16/36, 44%). One-quarter of participants (*n* = 17/68) reported that their families were unable to acquire a family support when needed over the previous 12 months.

**Table 4:** Family supports used and needed over previous 12 months

	Used *N* = 70 *n* (%)	Difficult to acquire^a^ *n*/*N* (%)	Did not receive enough *n*/*N* (%)	Unavailable when needed *N* = 70 *n* (%)
Any family support type	36 (51)	16/36 (44)	15/36 (42)	17 (25)
Specific family support type				
Counselling	15 (21)	6/15 (40)	7/15 (47)	7 (10)
Financial support	12 (17)	5/12 (42)	3/12 (25)	3 (4)
Respite services	10 (14)	5/10 (50)	5/10 (50)	6 (9)
Help to navigate non-health care systems (e.g. legal, governmental, insurance)	9 (13)	2/9 (22)	2/9 (22)	2 (3)
Education, skill building, or coping strategies	9 (13)	1/9 (11)	1/9 (11)	3 (4)
Advocacy for child’s needs	8 (11)	5/8 (63)	4/8 (50)	5 (7)
Caregiver support groups	7 (10)	0 (0)	1/7 (14)	2 (3)
Home care services	7 (10)	0 (0)	0 (0)	0 (0)
Care coordination services or comprehensive case management	5 (7)	0 (0)	2/5 (40)	2 (3)
Help navigating the health care system	5 (7)	3/5 (60)	3/5 (60)	2 (3)
Paid caregiver leave from work	4 (6)	0 (0)	1/4 (25)	5 (7)
Other^b^	4 (6)	1/4 (25)	0 (0)	1 (2)
*Missing*	*1*	--	--	*3*

^a^Grouping of ratings “very hard”, “somewhat hard”

^b^ ‘Other’ family supports used included: caregiving by extended family, unpaid caregiver leave from work, advice on care from on-call doctor, and Family Supports for Children with Disabilities. ‘Other’ family support not available: Drop-in childcare

### Impacts of pediatric IMDs for caregivers: employment, household finances, and caregiving-related QoL (objective 2)

More than one-quarter (*n* = 18, 27%) of participants reported 16 or more missed days of work among all of a child’s primary caregivers over the previous 12 months due to their child’s IMD; another ten participants (15%) reported 6 to 15 missed days (Table 5). 19% of participants reported that a primary caregiver had ever left or quit a job and 38% reported a primary caregiver had ever reduced work hours due to the child’s IMD. In our stratified analysis, there were no clear patterns among IMD trajectory and child age groups for missed days of work, leaving a job, or reducing hours, and our post-hoc inferential analysis identified no statistically significant differences.

**Table 5:** Employment and summary financial impacts of the inherited metabolic disease

	Full sample		Stratified by IMD trajectory and age group (< 5 vs. ≥ 5 years)												*p*-value^b^
			Chronic & non-progressive				Acute & episodic				Multi-system & progressive				
			< 5 y		≥ 5 y		< 5 y		≥ 5 y		< 5 y		≥ 5 y		
	*N*	*n* (%^a^)	*N*	*n* (%^a^)	*N*	*n* (%^a^)	*N*	*n* (%^a^)	*N*	*n* (%^a^)	*N*	*n* (%^a^)	*N*	*n* (%^a^)	
Missed days of work due to child’s IMD, past 12 months	70		16		7		14		15		7		11		
None		19 (28)		5 (31)		3 (43)		3 (23)		4 (31)		1 (14)		3 (27)	*0.28*
1–5 days		20 (30)		6 (38)		2 (29)		5 (38)		6 (46)		0 (0)		1 (9)	
6–15 days		10 (15)		2 (13)		2 (29)		2 (15)		0 (0)		2 (29)		2 (18)	
16 or more days		18 (27)		3 (19)		0 (0)		3 (23)		3 (23)		4 (57)		5 (45)	
* I don’t know*		*3*		*0*		*0*		*1*		*2*		*0*		*0*	
Ever left or quit a job because of child’s IMD, yes	70	13 (19)	16	1 (6)	7	2 (29)	14	1 (7)	15	4 (27)	7	2 (29)	11	3 (27)	*0.32*
Ever reduced job hours because of child’s IMD, yes^c^	69	26 (38)	16	4 (25)	7	2 (29)	13	3 (21)	15	7 (47)	7	4 (57)	11	6 (55)	*0.37*
Out-of-pocket expenses related to child’s IMD over past 12 months, yes^d, e^	68	42 (62)	16	9 (56)	7	5 (71)	14	5 (36)	13	7 (54)	7	5 (71)	11	11 (100)	*0.02*
Difficult to afford expenses related to child’s IMD, yes^f, g^	35	15 (43)	9	5 (56)	3	1 (33)	5	2 (40)	3	1 (33)	5	1 (20)	10	5 (50)	*0.73*

IMD = inherited metabolic disease

^a^ Valid percents presented

^b^ All statistical analyses compare six groups of combined child age and IMD category and were conducted using Fisher’s Exact test. Two-tailed *p*-values; α = 0.05

^c^ “I don’t know”: *n* = 1

^d^ “I don’t know”: *n* = 2

^e^ Expenses include products, medical foods/formulas, devices, supplies, equipment, household items and home modifications/renovations

^f^ Rated somewhat, very or extremely difficult

^g^ Missing: *n* = 3; “I prefer not to answer”: *n* = 4

More than half of families (*n* = 42, 62%) had incurred expenses related to their child’s IMD over the past 12 months (Table 5). When stratified by child age and IMD trajectory, our post-hoc analysis identified a statistically significant difference in the percentage of caregivers reporting out-of-pocket expenses, which ranged from 36% (5/14 caregivers to children < 5 years with acute and episodic IMDs) to 100% (11/11 caregivers to children ≥ 5 years with multi-system and progressive IMDs). Of those who reported having expenses, nearly half (43%) found it difficult to afford them (Table 5). 25% of participants reported out-of-pocket purchases of more than $1,000 in the past year. (Supplementary Table 3, Additional File 3).

**Fig. 2:**
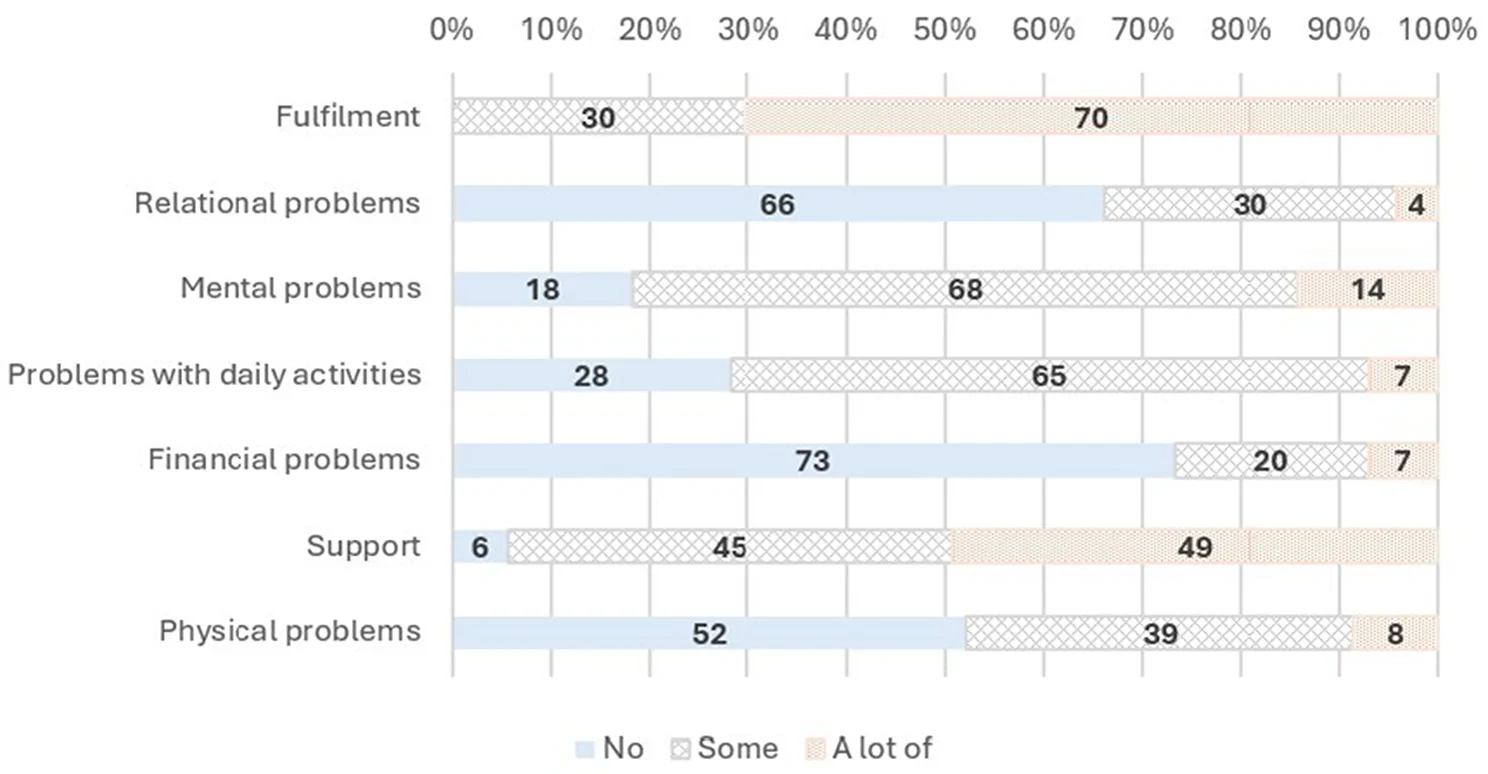
Caregiver quality of life, measured by the CarerQoL-7D (*N* = 71)

With respect to caregiving-related QoL, all caregivers reported ‘some’ or ‘a lot of’ fulfillment from their caregiving tasks based on the CarerQoL (Fig. 2), and the median score on the well-being question was 7.7 on a scale of 0 to 10 (Supplementary Table 4, Additional File 3). More than 80% of caregivers, however, reported ‘some’ or ‘a lot of’ mental problems, 72% reported ‘some’ or ‘a lot’ of problems with daily activities, and 51% reported ‘no’ or ‘some’ support for caregiving tasks (Fig. 2). There were no statistical differences in caregiving-related QoL when results were stratified by age and IMD trajectory (Supplementary Table 4, Additional File 3).

Also related to caregiver well-being, we considered the parental impact variables from our measures of caregiver-reported child HRQoL (Table 6). Among children < 5 years, those with a multi-system and progressive IMD had lower median scores (indicating higher impact) for the time impact of child health on caregivers (a difference of 25 points on scale of 0-100 relative to the other two groups, *p*-value = 0.03). Compared to population normative data, the mean emotional impact of child health on caregivers in our sample was more negative, with large effect sizes [77]: -1.68 and − 1.27 for caregivers of children ≥ 5 years and < 5 years, respectively (Supplementary Table 5, Additional File 3). Similarly, the mean impact of child health on caregiver time was more negative in our sample relative to population norms, with a large effect size among caregivers of children ≥ 5 years (-0.90) and a medium effect size among caregivers of children < 5 years (-0.72) (Supplementary Table 5, Additional File 3).

**Table 6:** Health-related quality of life of children with inherited metabolic disease (IMD), stratified by child age and IMD clinical trajectory

	All children in age category		Stratified by IMD clinical trajectory						*p*-value^a^
			Chronic & non-progressive		Acute & episodic		Multi-system & progressive		
	*N*	Median (Q1-Q3)	*N*	Median (Q1-Q3)	*N*	Median (Q1-Q3)	*N*	Median (Q1-Q3)	
**Children aged ≥ 5 years CHQ-PF50 subscale** ^b^									
^^Parental Impact – Emotional (PE)	34	45.8 (25.0–58.3)	7	58.3 (50.0–75.0)	15	41.7 (25.0–83.3)	12	37.5 (16.7–54.2)	0.16
^^Parental Impact – Time (PT)	34	66.7 (55.6–88.9)	7	88.9 (55.6–100)	15	66.7 (55.6–88.9)	12	66.7 (50.0–77.8)	0.38
Physical Functioning (PF)	34	91.7 (44.4–100)	7	100 (100–100)	15	100 (77.8–100)	12	41.7 (21.1–86.1)	0.001
Role/Social Limitations – Emotional/Behavioural (REB)	34	88.9 (55.6–100)	7	88.9 (55.6–100)	15	88.9 (44.4–100)	12	88.9 (50.0–100)	0.10
Role/Social Limitation – Physical (RP)	34	100 (33.3–100)	7	100 (100–100)	15	100 (66.7–100)	12	50.0 (25.0–100)	0.01
Bodily Pain/Discomfort (BP)	34	75.0 (50.0–80.0)	7	100 (80.0–100)	15	80.0 (60.0–80.0)	12	60.0 (45.0–60.0)	0.01
General Behaviour (BE)	34	73.8 (60.0–83.3)	7	64.2 (51.7–80.8)	15	75.0 (55.8–80.8)	12	80.0 (66.3–85.0)	0.23
Mental Health (MH)	34	70.0 (65.0–85.0)	7	70.0 (65.0–80.0)	15	70.0 (60.0–85.0)	12	72.5 (67.5–85.0)	0.83
Self-Esteem (SE)	34	77.1 (66.7–91.7)	7	91.7 (79.2–100)	15	75.0 (66.7–87.5)	12	70.8 (52.1–83.3)	0.10
General Health Perceptions (GH)	34	50.8 (30.8–64.2)	7	62.5 (60.0–66.7)	15	55.8 (39.2–66.7)	12	30.8 (20.0–43.3)	0.002
Family Activities (FA)	34	66.7 (45.8–83.3)	7	75.0 (54.2–83.3)	15	66.7 (50.0–95.0)	12	47.9 (33.3–72.9)	0.06
Family Cohesion (FC)	34	85.0 (60.0–85.0)	7	85.0 (60.0–85.0)	15	85.0 (60.0–85.0)	12	85.0 (60.0–92.5)	0.88
Physical Summary Measure (PhS)	34	46.2 (19.6–53.4)	7	53.4 (52.6–56.3)	15	48.4 (38.7–53.7)	12	16.6 (10.8–40.9)	0.001
Psychosocial Summary Measure (PsS)	34	46.0 (36.8–53.5)	7	48.1 (36.5–53.5)	15	43.1 (34.3–47.9)	12	48.7 (37.4–55.4)	0.65
**Children aged < 5 years** **ITQoL-SF47 subscale** ^b^									
^^Parental Impact – Emotional (PE)	37	91.7 (66.7–91.7)	16	78.1 (62.5–81.3)	14	78.1 (53.1–87.5)	7	81.3 (31.3–87.5)	0.98
^^Parental Impact – Time (PT)	37	85.0 (85.0–85.0)	16	91.7 (75.0–100)	14	91.7 (79.2–91.7)	7	66.7 (33.3–83.3)	0.03
Physical Abilities (PA)	37	94.4 (83.3–100)	16	93.9 (88.9–100)	14	93.9 (81.7–100)	7	94.4 (66.7–100)	0.96
Growth and Development (GD)	37	95.0 (85.0–100)	16	100 (85.0–100)	14	97.5 (92.5–100)	7	65.0 (50.0–90.0)	0.02
Bodily Pain/Discomfort (BP)	37	79.2 (75.0–87.5)	16	75.0 (75.0–75.0)	14	75.0 (62.5–93.8)	7	87.5 (75.0–100)	0.36
Temperament and Moods (TM)	37	68.8 (62.5–87.5)	16	79.2 (70.0–87.5)	14	83.3 (75.0–89.6)	7	79.2 (79.2–91.7)	0.54
General Behaviour (GB)^c^	27	77.9 (68.8–86.3)	9	62.5 (50.0–87.5)	11	75.0 (62.5–87.5)	7	75.0 (37.5–93.8)	0.54
Combined Behaviour (CBE)^c^	27	60.0 (47.5–70.8)	9	71.7 (65.4–81.3)	11	80.0 (73.8–90.4)	7	79.2 (54.6–86.3)	0.25
General Health Perceptions (GH)	37	81.3 (62.5–87.5)	16	55.8 (47.5–70.8)	14	64.2 (57.1–72.9)	7	55.8 (26.7–60.0)	0.19
Family Cohesion (FC)	36	94.4 (83.3–100)	15	93.9 (88.9–100)	14	85.0 (85.0–85.0)	7	85.0 (60.0–85.0)	0.32

IMD = inherited metabolic disease

^a^ Krustkal-Wallis *H* test, for comparison of median scores stratified by IMD clinical trajectory

^b^ Higher scores represent higher HRQoL for all subscales

^c^ Scale questions answered only by parents of children aged 1 or older

^^ Reported with results on caregiving-related QoL

### Child health-related quality of life (objective 3)

Although sample sizes by IMD clinical course trajectory were small, among children aged ≥ 5 years in our study, those with multi-system and progressive IMDs had lower median scores than children with other IMD clinical trajectories on most subscales (Table 6), with the highest absolute difference in medians (> 20 points on scales of 0-100) and a *p*-value of < 0.05 observed for: Physical Functioning, Role/Social Limitation – Physical, Bodily Pain/Discomfort, General Health Perceptions, and the Physical Summary Measure. In comparing our sample to population normative scores (Supplementary Table 5, Additional File 3), children aged ≥ 5 years in our study had lower mean HRQoL scores across both summary measures and all subscales except Family Cohesion, and many effect sizes were ‘large’ (absolute value > 0.8), per Cohen’s guidelines [77]. The largest effect sizes were observed in the Physical Summary Measure (-1.41) and the General Health Perceptions and Family Activities subscales (each − 1.38).

Among children < 5 years in our sample, the median scores for most HRQoL subscales did not differ significantly depending on IMD clinical trajectory. Those with multi-system and progressive IMDs had a lower median score than children with other IMD clinical trajectories on the Growth and Development subscale (a difference of 32.5 points on a scale of 0-100, *p*-value = 0.02) (Table 6). Compared to population norms, younger children in our sample had lower mean scores on all subscales except Family Cohesion (Supplementary Table 5, Additional File 3). Most effect sizes were medium (absolute value 0.5–0.8) but the effect size for General Health Perceptions was large (-1.46). There were no trends of differences in child HRQoL by child sex for either age group (Supplementary Table 6, Additional File 3).

## Discussion

### Summary of findings

We described caregivers’ reported experiences with managing care for their children with IMDs and the impacts of that care management on family time, finances, and quality of life. From our exploratory analyses, children’s care needs and their associated impacts on families appeared to differ based on the clinical trajectory of a child’s specific IMD diagnosis: caregivers to children with diagnoses considered to be progressive and with multi-system involvement were more likely to report out-of-pocket expenses and lower child HRQoL.

### The unmet needs of caregivers

Our study highlights potential unmet needs of caregivers of young children with IMDs in the areas of caregiver time, healthcare responsibilities and psychological or emotional health. For example, our results collectively demonstrate that IMDs have a consequential effect on caregivers’ time and quality of life. Participants reported using care supports and interventions that can alleviate the impacts of caregiving (e.g., support services), as well as those that require families to manage care (e.g., diet, medication). We estimated, conservatively, that close to half of families spent a minimum of 4 h per week, and 15% spent a minimum of 8 h per week, or the equivalent of a standard workday, on IMD care beyond general child caregiving tasks. Additionally, we observed lower scores (indicating more impact) on the HRQoL domain of parental time relative to population norms for both children < 5 and ≥ 5 years. With this time commitment to disease care, it may not be surprising that more than one-third of participants reported that a primary caregiver had reduced employment hours due to their child’s IMD. Most studies that have attempted to quantify the time spent by caregivers of children on IMD care management have focused on dietary management for one specific IMD, phenylketonuria [39–42]. These studies estimated a time consumption of approximately 7 to 19 h per week for diet management [39, 41, 42]. Measurement methods and time periods across studies varied, making it difficult to compare to our findings. The high proportion of participants in our study who reported caregiving impacts on employment is consistent with many other studies where caregivers, especially mothers, have reported quitting jobs, reducing work hours, switching to a less demanding job, or giving up job opportunities to care for their child with an IMD [32, 36, 42–44, 78, 79]. There may have been additional household factors that affected the impact of the IMD on family time but which we were unable to analyze due to small cell sizes. For example, studies suggest that single- or divorced-parent households of children with chronic conditions (of which there were five in this study) carry more illness-related responsibility [80–82]. Families with multiple children with an IMD (of which our study included seven) may also experience additional time required to provide IMD care. Also, policies implemented to control the spread of COVID-19 may have exacerbated family IMD care responsibilities: approximately 25% of caregivers in our larger cohort study reported pandemic-related effects on IMD care responsibilities and 30% reported effects on caregiver employment, respectively [83]. 

In addition to IMD impacts on caregiver time, most caregivers in our study reported at least some mental health struggles. Similarly, we identified emotional impacts on caregivers as an area where participants in our study had lower scores (i.e., lower QoL) relative to population norms. Consistent with our study, several cross-sectional studies using validated instruments have found that caregivers of children with a range of IMDs experience poor emotional health, stress, anxiety and/or depression, and have inadequate time for daily activities [30, 31, 34, 36, 84]. All study participants also, however, reported the caregiving role to be at least somewhat fulfilling despite its intensity. Studies of caregivers of children with disabilities have also found that they often experience happiness, purpose, and personal growth [85, 86]. 

Caregivers of children with IMDs may benefit from greater access to psychological and social supports. Among our study participants, use of family supports such as counselling was not widespread; only about half reported use of family supports over the previous 12 months, and 25–50% of those caregivers felt that they received an inadequate amount of those supports. A study by Pelentsov and colleagues similarly found that parents of children with rare disease were often not offered, or did not know how to access, available psychological support services [87]. Psychological supports that address adaptive coping strategies, which have been associated with better caregiver psychological QoL [88], may help to address the psychological needs of caregivers of young children with IMDs. While taking into account the additional challenges that caregivers of children with multi-system and progressive IMDs may face, an approach that considers and builds on family strengths alongside provision of additional supports may be an important component of effective interventions to support families of children with IMDs [89, 90]. In addition, healthcare policies and interventions that reduce the substantial responsibility that caregivers carry and provide flexibility for workforce participation, should be considered. Social care, such as the assistance of a social worker, can help families with dietary management, access to support services, and psychological support [91]. Previous research suggests that care coordination models and services provided virtually, by increasing access to needed resources, may have positive effects on psychological health, and that respite care may provide emotional and physical relief to families of children with rare or complex conditions [92, 93]. Caregiver benefits and workplace accommodations could provide family caregivers with more opportunity to pursue paid employment, if desired [92, 94]. An important direction for future studies would be an examination of the policies and supports that would be most beneficial to caregivers of children with IMDs, taking into consideration key child, caregiver, and family characteristics.

### Child quality of life and support for IMD care

In our study, caregivers reported that children with IMDs had lower HRQoL on most domains compared to children in the general population, and we found significant differences in several HRQoL domains by IMD clinical trajectory for children aged ≥ 5 years, particularly in physical domains, with lowest scores for children with multi-system and progressive IMDs. Various IMDs may manifest a broad spectrum of clinical symptoms; although children with IMDs are usually followed by metabolic and genetic specialists, the clinical course trajectories that we have used to categorize IMDs eligible for this study are characterized by different healthcare needs. For example, phenylketonuria (PKU) is a chronic and non-progressive IMD that has historically required lifelong dietary intervention to maintain metabolic control of phenylalanine levels but is manageable with early diagnosis and treatment initiation [95]. Medium-chain acyl-coenzyme A dehydrogenase deficiency (MCAD deficiency) is an acute and episodic IMD characterized by risk of metabolic decompensation triggered by prolonged fasting, where children under two years old seek acute care at much higher rates than the general population but often experience a decline in acute care visits as they get older [96]. Comparatively, children with multi-system and progressive IMDs often experience intensive physical symptoms and sometimes neurodevelopmental manifestations that worsen over the lifespan. For example, mucopolysaccharidoses (MPS), a group of lysosomal storage disorders, are often characterized by skeletal, cardiac, and respiratory issues, progressive organ damage, pain, and restricted mobility. Some MPS subtypes are also neuronopathic; cognitive decline may lead to a significant increase in family care responsibilities [97].

Few studies have compared HRQoL scores between children with different IMDs. Bosch and colleagues reported that children with “acute” intoxication-type IMDs, such as maple syrup urine disease, had significantly lower overall HRQoL, psychosocial health, physical function, and social function than children with PKU [5]. We did not observe such differences in our study, but the groups that we compared were not composed of the same IMDs. Although the CHQ-PF50 and ITQoL-SF47 have been used in other studies of children with IMDs, including progressive conditions such as MPS [12, 13, 15, 98–104], a challenge in interpreting our findings is that generic instruments may not capture the most important dimensions of HRQoL in specific conditions, particularly progressive conditions, over time [105–108]. The rarity of IMDs poses a challenge to developing disease-specific instruments, although a few have been developed [109–111]. Research investigating whether there are dimensions of HRQoL that are important to capture but missing from validated but generic tools, alongside longitudinal research on the determinants of child HRQoL (which was beyond the capacity of this cross-sectional study) would better support targeted care interventions to improve HRQoL for this population [59].

Among caregivers who reported not receiving adequate supports or interventions for their child’s IMD, a common concern was inadequate accommodations from schools, and a lack of some therapeutic and social services. Due to small numbers, we were unable to assess factors associated with inadequate levels of supports. Previous research on children with rare diseases and children with medical complexity have identified perceived challenges including: insufficient funding or insurance coverage, turnover among care support workers, and support workers who lack the skills to properly care for a rare or complex condition [112–116]. Improving the levels of care supports would decrease the responsibility for IMD care placed on family caregivers, which may result in lower stress levels and reduction in burnout [92, 116], and would be critical to facilitating equitable opportunities for children with IMDs to participate in daily activities.

Our data were collected during the COVID-19 pandemic, after new public health policies and practices across Canada were implemented in response to the pandemic. This may have affected access to support services, increased the level of responsibility caregivers assumed for their child’s care, and influenced both caregiver and child HRQoL. However, challenges to healthcare in educational systems [34, 50, 117], impaired HRQoL for children with IMDs compared to normative populations [4–7, 9, 118], and emotional challenges for caregivers had all been described prior to the pandemic [30–34, 36, 38, 43, 84, 119, 120], suggesting that these challenges are longstanding.

### Strengths and limitations

Our results highlight current experiences and challenges faced by caregivers of children with IMDs: difficulties managing IMD care at home, an average minimum of four additional hours per week for care management beyond general childcare, with many caregivers spending much more time, and impacts of IMD care on paid employment and on caregiver and child HRQoL. We recruited participants from across Canada and comprehensively assessed their experiences with caring for children with IMDs. We had high consent and data completion rates.

This study has limitations. Our relatively small sample limited our ability to explore the impact of some variables such as caring for multiple children with an IMD, and to make strong comparisons across IMD diagnostic trajectories. We did not have a priori hypotheses regarding the relationship between variables, so results of our inferential analyses should be interpreted with caution. Due to the length and complexity of the cohort study questionnaires, we offered participation only in English. In addition, our sample may not have been representative of the target population. More than half of participants had at least an undergraduate university degree, compared to 33% of Canadians aged 25 to 64 years [121]. Half of participants had a pre-tax annual household income of >$100,000, while the national median after-tax annual household income in 2021 was $68,400 [122]. Children and families who may need additional supports as part of their care, including but not limited to financial and language supports, were thus likely under-represented, affecting the generalizability of our findings. Caregivers whose children were experiencing urgent or critical health care issues, whose children were newly diagnosed (often associated with younger age), who have disabilities themselves, or who were experiencing significant financial and time costs may have felt overwhelmed and been less likely to participate in our study than caregivers whose own health or children’s health issues were relatively stable [50, 123]. As household income, parental educational attainment, recent immigration (particularly when co-occurring with language barriers), and higher levels of responsibility for care may be associated with lower HRQoL, families under-represented in this study may experience even greater challenges to accessing and managing care, and reduced HRQoL, than our study participants [18, 124–127].

We prioritized the selection of participants who expected the designated child to have multiple health care encounters during the study; our sample was therefore over-representative of families who are frequent healthcare users.

Although most respondents in our study identified as mothers, we did not collect data specifically on caregiver gender and thus were not able to examine the effects of gender on caregiver needs or experiences. Given previous research on the gender differences in caregiving activities, and the gendered effects on the psychological health of caregivers of children with chronic illnesses, investigating the impact of gender on caring for a child with an IMD may be an interesting direction for future research [128–132]. Finally, we ascertained needs from caregivers’ perspectives due to the young age of their children; future research would benefit from the perspectives of children and youth themselves.

## Conclusion

Families of children with IMDs assume substantial responsibility for the management of IMD care at home and on a daily basis, particularly around the monitoring and preparation of diets and administration of supports and interventions. Caregivers in our study are spending a minimum of 4 h per week on IMD care on average, and report impairment to both caregiver and child HRQoL. Although exploratory, our findings also suggest that there is heterogeneity by child age and IMD trajectory in both IMD care management and caregiver/child HRQoL. Future research should examine the specific unmet care needs in these sub-populations to build and deliver effective supports to children with IMDs and their families. Relief from high levels of caregiving responsibility, time, and cost should be a priority for healthcare policies and supportive interventions. Such supports would likely have an appreciable impact on psychological and physical health outcomes for both children with IMDs and their caregivers.

## Supplementary Information

Below is the link to the electronic supplementary material.

Supplementary Material 1: Additional File 1. Eligible inherited metabolic diseases.

Supplementary Material 2: Additional File 2. Key team-developed questions and response options.

Supplementary Material 3: Additional File 3. Additional results.

## Data Availability

The study questionnaire is available from the corresponding author upon reasonable request.
