# Supplementary material for: The impacts of caring for children with inherited metabolic diseases for families: a cross-sectional study
Source: Orphanet J Rare Dis. 2026 Jun 10;21:286. doi: 10.1186/s13023-026-04434-y (PMC13487924; doi:10.1186/s13023-026-04434-y)
Supplement: Supplementary file 1 [file 13023_2026_4434_MOESM1_ESM.docx]

**Additional File 1: Eligible inherited metabolic diseases**

| ***Typical IMD clinical course trajectory*** | | |
| --- | --- | --- |
| **Chronic and non-progressive disease** | **Diseases characterized by acute episodes of severe illness** | **Progressive multi-system disease** |
| Carnitine uptake defect  Citrin deficiency  Glutaric acidemia type I  Galactosemia  Guanidinoacetate methyltransferase deficiency  Homocystinuria / CBS deficiency  Multiple carboxylase/ biotinidase deficiency  Phenylalanine hydroxylase deficiency  Pyridoxine-dependent epilepsy  Tyrosinemia type I | Arginase deficiency  Argininosuccinic aciduria  Beta-Ketothiolase (BKT) deficiency  Carbamoyl phosphate synthetase deficiency  Citrullinemia  Glycogen storage disease type 1  Hyperornithinemia-Hyperammonemia-Homocitrullinuria syndrome  HMG-CoA lyase Deficiency  Isovaleric acidemia (IVA)  Long-chain 3-hydroxyacyl-CoA dehydrogenase (LCHAD) deficiency  Maple syrup urine disease  Medium chain acyl-CoA dehydrogenase (MCAD) deficiency  Methylmalonic acidemias  N-acetylglutamate synthetase deficiency  Ornithine transcarbamylase deficiency  Propionic acidemia  Trifunctional protein deficiency  VLCAD deficiency | Farber disease  Mucopolysaccharidosis Type I  IMDs that: (1) involve 3 or more organ systems and (2) chronic complications of the disease get progressively worse over time, even with available treatment*  ** IMDs were not specified in advance but centres used these criteria to identify eligible participants. Among study participants, there were thirteen different IMDs included in this trajectory.* |
