# Supplementary material for: The impacts of caring for children with inherited metabolic diseases for families: a cross-sectional study
Source: Orphanet J Rare Dis. 2026 Jun 10;21:286. doi: 10.1186/s13023-026-04434-y (PMC13487924; doi:10.1186/s13023-026-04434-y)
Supplement: Supplementary file 2 [file 13023_2026_4434_MOESM2_ESM.docx]

**Additional File 2: Key team-developed questions and response options**

| **Question** | **Response options** | |
| --- | --- | --- |
| Do you have a plan, protocol or written directions from the metabolic clinic for managing your participating child's metabolic disease (e.g. a sick day protocol)? | Yes   No   I don't know | |
| Over the past 12 months, what types of treatments, therapies, services, products or equipment have you used to manage your child's inherited metabolic disease?   Check ALL that apply. | Prescription drugs or medication   Medical foods or formulations   Dietary supplements (not foods or formulations)   Dietary recommendations or specifications from the metabolic doctor or dietitian   Dietary support services (e.g. feeding clinic)   Exercise prescription or recommendation from doctor   Exercise or physical support services (eg physiotherapy)   Supplemental oxygen therapy   Home care   School assistance or accommodations   Counselling or mental health support for child   Neuropsychological or cognitive assessments   Respite care   Devices or equipment (e.g., feeding tube, mobility aid)   Early childhood interventions   Transportation services   Social services (e.g., social worker)   Home modifications   Other products (specify: __________)   Other services or therapies (specify: __________)   None of the above | |
| *If checked ‘devices or equipment’ above:*  Specify what devices and equipment you have used over the past 12 months to manage your participating child's inherited metabolic disease.  Check ALL that apply. | Feeding tube or device   Splint   Mobility aid   Ventilator   Continuous positive airway pressure therapy (CPAP)   Wheelchair   Corrective lenses   Hearing aid   Glucometer   Other (specify: __________) | |
| Over the past 12 months, were there services, therapies or products that your child needed to manage their IMD that you could not get WHEN they needed it?   Check ALL that apply. | Prescription drugs or medication   Medical foods or formulations   Dietary supplements (not foods or formulations)   Dietary recommendations or specifications from the metabolic doctor or dietitian   Dietary support services (e.g. feeding clinic)   Exercise prescription or recommendation from doctor   Exercise or physical support services (eg physiotherapy)   Supplemental oxygen therapy   Home care   School assistance or accommodations   Counselling or mental health support for child   Neuropsychological or cognitive assessments   Respite care   Devices or equipment (e.g., feeding tube, mobility aid)   Early childhood interventions   Transportation services   Social services (e.g., social worker)   Home modifications   Other products (specify: __________)   Other services or therapies (specify: __________)   None of the above | |
| *For each service, therapy or product checked:* Why were the necessary [service, therapy, product] not available when needed? Were the reasons: | Financial   Non-financial   Both | |
| *If financial:* Please specify the FINANCIAL reasons why the [service, therapy, product] were not available.  Check all that apply. | They were not covered by public health care   They were not covered by our private health insurance   We did not have private health insurance   We could not afford to pay for them   Other financial reason(s) (specify: __________)   I prefer not to answer | |
| *If non-financial:* Please specify the NON-FINANCIAL reasons why the [service, therapy, product] were not available.  Check all that apply. | They were not available in our province / territory   They were available but we would have had to travel too far to receive them   There was a waiting list to receive them   My child's doctor would not write a prescription   Other non-financial reason(s) (specify: __________) | |
| *For each of the types of treatments, therapies, services, products or equipment used to manage child's IMD over past 12 months:* Over the past 12 months, how hard was it to get [service, therapy, product]? | Very easy   Somewhat easy   Somewhat hard   Very hard | |
| *For each of the types of treatments, therapies, services, products or equipment used to manage child's IMD over past 12 months:* Over the past 12 months, did you get ENOUGH of [service, therapy, product]? | Got enough   Did NOT get enough | |
| *For types of treatments, therapies, services, products or equipment used to manage child's IMD over past 12 months (prescription drug regimen, diet control, exercise regimen, supplemental oxygen therapy, feeding tube or device, splint, mobility aid, ventilator, CPAP, wheelchair, corrective lenses, hearing aid, glucometer, "other"):* How difficult is it for you to manage [service, therapy, product] of your child's care at home? | Not difficult   Somewhat difficult   Very difficult   Extremely difficult   Not used at home   I don't know | |
| *If checked medical foods or formulations, dietary supplements (not foods or formulations), or dietary recommendations or specifications from the metabolic doctor or dietitian used over past 12 months:* Does your family need to spend extra time planning and preparing special meals because of your child's inherited metabolic disease? | Yes   No   I don't know | |
| *If yes, need extra time planning/preparing special meals:* How much EXTRA time per WEEK on average does your family spend planning and preparing meals because of your child's inherited metabolic disease? | 1 hour or less per week   More than 1 hour and up to 4 hours   More than 4 hours and up to 7 hours   More than 7 hours and up to 10 hours   More than 10 hours and up to 14 hours   More than 14 hours   I don't know | |
| *For treatments, therapies, services, products or equipment used to manage child's IMD over past 12 months (prescription drug regimen, exercise regimen, supplemental oxygen therapy, feeding tube or device, splint, mobility aid, ventilator, CPAP, wheelchair, corrective lenses, hearing aid, glucometer, "other"):* How much time PER WEEK on average does your family spend helping your child with [device, therapy]? | None   1 hour or less   More than 1 hour & up to 2 hours   More than 2 hours & up to 3 hours   More than 3 hours & up to 4 hours   More than 4 hours | |
| Over the past 12 months what type of support services for FAMILY MEMBERS have you used?  Check ALL that apply. | Counselling   Caregiver support groups (in-person or online)   Crisis support   Financial support   Respite services   Home visit   Care coordination services or comprehensive case management   Help to navigate the health care system   Help to navigate other systems (e.g. legal, governmental, insurance)   Advocacy for your child's needs   Education, skill building or coping strategies   Paid caregivers' leave from work   Other (specify: __________)   None of the above | |
| *For each type of family support service checked:* Over the past 12 months, how hard was it to get [support service for family member]? | Very easy   Somewhat easy   Somewhat hard   Very hard | |
| *For each type of family support service checked:* Over the past 12 months, did you get ENOUGH of [support service for family member]? | Got enough   Did NOT get enough | |
| Over the past 12 months, are there family support services that your family needed that you could not get WHEN you needed it?   Check ALL that apply. | Counselling   Caregiver support groups (in-person or online)   Crisis support   Financial support   Respite services   Home visit   Care coordination services or comprehensive case management   Help to navigate the health care system   Help to navigate other systems (e.g. legal, governmental, insurance)   Advocacy for your child's needs   Education, skill building or coping strategies   Paid caregivers' leave from work   Other (specify: __________)   None of the above | |
| Over the past 12 months, how many DAYS in TOTAL have your child's primary caregivers missed paid work due to caring for your participating child, for any reason? | None   1 - 3 days   4 - 5 days   6 - 10 days   11 - 15 days | 16 - 20 days   21 - 25 days   26 days or more   I don't know |
| Have any of your child's primary caregivers ever LEFT or QUIT a job because of your child's inherited metabolic disease? | Yes   No | |
| Have any of your child's primary caregivers ever had to REDUCE their paid work hours because of your child's inherited metabolic disease? | Yes   No   I don't know | |
| Over the past 12 months, did you have to buy any products (including medical foods and formulas), devices, supplies, equipment or household items in order to manage your child's inherited metabolic disease? | Yes   No   I don't know | |
| *If yes, had to buy products to manage child’s IMD:* How much, in Canadian dollars, did your family pay out of pocket to buy these products in the past 12 months? | $200 or less   $201 to $400   $401 to $600   $601 to $800   $801 to $1,000   $1,001 to $1,200 | $1,201 to $1,400   $1,401 to $1,600   $1,601 to $1,800   $1,801 to $2,000   $2,001 or more   I don't know |
| Over the past 12 months, did you need to make any permanent or temporary modifications or renovations to your home to accommodate your child's inherited metabolic disease? | Yes   No | |
| *If yes, had to modify/renovate home to accommodate child’s IMD:* How much, in Canadian dollars, did your family pay out of pocket to modify or renovate your home in the past 12 months to accommodate your child’s inherited metabolic disease? | $1,000 or less   $1,001 to $5,000   $5,001 to $10,000   $10,001 to $20,000   $20,001 to $30,000 | $30,001 to $40,000   $40,001 to $50,000   $50,001 or more   I don’t know |
| *If yes, had to buy products or modify/renovate home:* How difficult was it for your family to afford these recent costs (home modifications and/or products)? | Not at all difficult   Somewhat difficult   Very difficult   Extremely difficult   I prefer not to answer | |
