# Supplementary material for: The impacts of caring for children with inherited metabolic diseases for families: a cross-sectional study
Source: Orphanet J Rare Dis. 2026 Jun 10;21:286. doi: 10.1186/s13023-026-04434-y (PMC13487924; doi:10.1186/s13023-026-04434-y)
Supplement: Supplementary file 3 [file 13023_2026_4434_MOESM3_ESM.docx]

**Additional File 3: Additional results**

**Supplementary Table 1.** Descriptive statistics, IMD trajectory categories by child age

|  | **Full sample** | **IMD trajectory** | | | |
| --- | --- | --- | --- | --- | --- |
|  |  | Chronic & non-progressive | Acute &  episodic | Multi-system & progressive | |
|  | N =71 | N =23 | N=29 | N=19 | |
|  |  | **n (%)** | | | |
| Child age, y |  |  |  |  | |
| 0 – 1 | 18 | 11 (48) | 6 (21) | 1 (5) | |
| 2 – 3 | 10 | 3 (13) | 4 (14) | 3 (16) | |
| 4 – 5 | 13 | 4 (17) | 6 (21) | 3 (16) | |
| 6 – 7 | 10 | 3 (13) | 3 (10) | 4 (21) | |
| 8 – 9 | 13 | 1 (4) | 6 (21) | 6 (32) | |
| 10 – 11 | 5 | 0 (0) | 4 (14) | 1 (5) | |
| 12 - 13 | 2 | 1 (4) | 0 (0) | 1 (5) | |
|  | | **Median (Q1 – Q3)** | | | |
| Child age, y | | 2.0 (0.0 - 5.0) | 5.0 (2.0 - 8.0) | | 7.0 (4.0 - 8.0) |

**Supplementary Table 2.** Additional data on IMD care supports and interventions

|  | n (%) |
| --- | --- |
| Care supports/interventions unavailable when needed, count (N=71) |  |
| More than one | 10 (15) |
| One | 10 (15) |
| None | 51 (70) |
| *Missing* | *5* |
| Care supports/interventions unavailable when needed, type (N=71) |  |
| School assistance or accommodation | 9 (14) |
| Medical foods/formulas | 5 (8) |
| Dietary supplements | 3 (5) |
| Early childhood interventions | 3 (5) |
| Exercise or physical support services | 3 (5) |
| Prescription drugs/medication | 3 (5) |
| Counselling or mental health support | 2 (3) |
| Home care | 2 (3) |
| Neuropsychological or cognitive assessments | 2 (3) |
| Respite care | 2 (3) |
| Other^a^ | 3 (5) |
| *Missing* | *5* |
| Perceived reasons for unavailable items (N=20) |  |
| Waiting list | 13 (65) |
| Not available in home province/territory | 11 (55) |
| Not covered by public health care | 3 (15) |
| Other – COVID (general or unspecified) | 6 (30) |
| Other – shortages | 3 (15) |
| Other^b^ | 6 (30) |
| ^a^Includes social services, neoheel lancets, suction catheters  ^b^Includes: not covered by private insurance, do not have private insurance, could not afford it, too far away | |

**Supplementary Table 3.** Detailed financial impacts of inherited metabolic disease (IMD) care on the family (N=71)

|  | n (%) |  |
| --- | --- | --- |
| Out-of-pocket purchases^a^ to manage child’s IMD over previous 12 months |  |  |
| No purchases | 26 (41) |  |
| $1 - $200 | 7 (11) |  |
| $201 - $1,000 | 14 (22) |  |
| More than $1,000 | 16 (25) |  |
| *I don’t know* | *5* |  |
| *Missing* | *3* |  |
| Out-of-pocket expenses to modify/renovate the home over previous 12 months to accommodate child’s IMD |  |  |
| No expenses | 65 (94) |  |
| $1,000 or less | 4 (6) |  |
| *I don’t know* | *1* |  |
| *Missing* | *1* |  |
| ^a^ For products, medical foods/formulas, devices, supplies, equipment, household items | | |

| **Supplementary Table 4.** Caregiver quality of life, stratified by inherited metabolic disease trajectory and age group | | | | | | | | | | | | | | |
| --- | --- | --- | --- | --- | --- | --- | --- | --- | --- | --- | --- | --- | --- | --- |
|  |  | **CarerQoL score^a^** | | | | | | | | | | | |  |
|  |  | **Fulfilment** | | | **Relational problems** | | | **Mental problems** | | | **Problems with daily activities** | | |  |
|  | N | *No* | *Some* | *A lot* | *No* | *Some* | *A lot* | *No* | *Some* | *A lot* | *No* | *Some* | *A lot* |  |
|  |  | n (%) | | | | | | | | | | | |  |
| Full sample | 71 | 0 (0) | 21 (30) | 50 (70) | 47 (66) | 21 (30) | 3 (4) | 13 (18) | 48 (68) | 10 (14) | 20 (28) | 46 (65) | 5 (7) |  |
| **IMD trajectory, age group (years)** |  |  |  |  |  |  |  |  |  |  |  |  |  |  |
| Chronic & non-progressive, <5 | 16 | 0 (0) | 2 (13) | 14 (88) | 14 (88) | 2 (13) | 0 (0) | 13 (18) | 11 (73) | 1 (7) | 5 (33) | 11 (69) | 0 (0) |  |
| Chronic & non-progressive, ≥5 | 7 | 0 (0) | 3 (43) | 4 (57) | 5 (71) | 2 (29) | 0 (0) | 4 (25) | 4 (57) | 1 (14) | 2 (29) | 4 (57) | 1 (14) |  |
| Acute & episodic, <5 | 14 | 0 (0) | 4 (29) | 10 (71) | 7 (50) | 7 (50) | 0 (0) | 2 (29) | 10 (71) | 3 (21) | 4 (29) | 10 (71) | 0 (0) |  |
| Acute & episodic, ≥5 | 15 | 0 (0) | 6 (40) | 9 (60) | 8 (53) | 5 (33) | 2 (13) | 1 (7) | 9 (60) | 3 (20) | 8 (53) | 6 (40) | 1 (7) |  |
| Multi-system & progressive, <5 | 7 | 0 (0) | 3 (38) | 4 (57) | 5 (71) | 2 (29) | 0 (0) | 3 (20) | 6 (75) | 0 (0) | 0 (0) | 6 (86) | 1 (13) |  |
| Multi-system & progressive, ≥5 | 12 | 0 (0) | 3 (25) | 9 (75) | 8 (67) | 3 (25) | 1 (8) | 1 (14) | 8 (67) | 2 (17) | 1 (8) | 9 (75) | 2 (17) |  |
| *p-value*^b^ |  | *0.46* | | | *0.37* | | | *0.91* | | | *0.09* | | |  |
|  |  |  |  |  |  |  |  |  |  |  |  |  |  |  |
|  |  | **CarerQoL score^a^** | | | | | | | | | | | |  |
|  |  | **Financial problems** | | | **Support** | | | **Physical problems** | | | **Well-being** | | |  |
|  | N | *No* | *Some* | *A lot* | *No* | *Some* | *A lot* | *No* | *Some* | *A lot* |  |  |  |  |
|  |  | n (%) | | | | | | | | | Median (Q1 – Q3) | | |  |
| Full sample | 71 | 52 (73) | 14 (20) | 5 (7) | 4 (6) | 32 (45) | 35 (49) | 37 (52) | 28 (39) | 6 (8) | 7.7 (7.0 – 8.8)^c^ | | |  |
| **IMD trajectory, age group (years)** |  |  |  |  |  |  |  |  |  |  |  | | |  |
| Chronic & non-progressive, <5 | 15 | 12 (75) | 3 (20) | 1 (7) | 2 (13) | 7 (47) | 7 (44) | 10 (63) | 5 (33) | 1 (7) | 8.0 (7.0 – 8.9) | | |  |
| Chronic & non-progressive, ≥5 | 7 | 5 (71) | 2 (29) | 0 (0) | 0 (0) | 3 (43) | 4 (57) | 4 (57) | 3 (43) | 0 (0) | 7.5 (6.8 - 8.2) | | |  |
| Acute & episodic, <5 | 14 | 11 (79) | 3 (21) | 0 (0) | 2 (14) | 2 (14) | 10 (71) | 6 (43) | 7 (50) | 1 (7) | 8.1 (7.5 - 9.0) | | |  |
| Acute & episodic, ≥5 | 15 | 12 (80) | 2 (13) | 1 (7) | 0 (0) | 8 (53) | 7 (47) | 7 (47) | 6 (40) | 2 (13) | 8.0 (7.0 - 8.8)^d^ | | |  |
| Multi-system & progressive, <5 | 8 | 4 (57) | 2 (25) | 1 (13) | 0 (0) | 4 (50) | 3 (43) | 4 (57) | 2 (25) | 1 (13) | 7.0 (5.0 - 8.0) | | |  |
| Multi-system & progressive, ≥5 | 12 | 8 (67) | 2 (17) | 2 (17) | 0 (0) | 8 (67) | 4 (33) | 6 (50) | 5 (42) | 1 (8) | 7.5 (6.6 - 8.3) | | |  |
| *p-value* |  | *0.91* | | | *0.23* | | | *0.99* | | | *0.49* | | |  |
| ^a^ Fulfilment, relational problems, mental problems, problems with daily activities, financial problems, support, and physical problems measured using the CarerQoL-F.^66-68^ Happiness measured using the CarerQoL-VAS, scale 0 (‘completely unhappy’) – 10 (‘completely happy’).^66-68^  ^b^ All statistical analyses conducted using Fisher’s Exact test except for Well-Being domain which was conducted using the Kruskal-Wallis *H* test. Two-tailed *p*-values; α = 0.05.  ^c^ N = 70  ^d^ N = 14 | | | | | | | | | | | | | | |

**Supplementary Table 5.** Health-related quality of life scores of children with inherited metabolic disease compared to population norm samples

|  | **N** | **Study sub-sample** | **Population norm** | **Effect size**  **Hedges’ *g***^a^ |
| --- | --- | --- | --- | --- |
|  |  | Mean (SD) | Mean (SD) |  |
| **Children aged ≥5 years**  **CHQ-PF50 subscale**^b^ |  |  |  |  |
| ^^Parental Impact – Emotional (PE) | 34 | 47.06 (26.42) | 80.3 (19.1) | - 1.68 ^d^ |
| ^^Parental Impact – Time (PT) | 34 | 69.61 (22.44) | 87.8 (19.9) | - 0.90 ^d^ |
|  |  |  |  |  |
| Physical Functioning (PF) | 34 | 75.42 (32.68) | 96.1 (13.9) | - 1.28^d^ |
| Role/Social Limitations – Emotional/Behavoural (REB) | 34 | 75.49 (30.56) | 92.5 (18.6) | - 0.86 ^d^ |
| Role/Social Limitation – Physical (RP) | 34 | 75.00 (34.39) | 93.6 (18.6) | - 0.92 ^d^ |
| Bodily Pain/Discomfort (BP) | 34 | 70.29 (22.36) | 81.7 (19.0) | - 0.59 ^e^ |
| Behaviour (BE) | 34 | 69.90 (16.51) | 75.6 (16.7) | - 0.34 |
| Mental Health (MH) | 34 | 70.59 (15.94) | 78.5 (13.2) | - 0.59 ^e^ |
| Self-Esteem (SE) | 34 | 75.49 (19.29) | 79.8 (17.5) | - 0.24 |
| General Health Perceptions (GH) | 34 | 48.70 (20.72) | 73.0 (17.3) | - 1.38 ^d^ |
| Family Activities (FA) | 34 | 63.58 (22.75) | 89.7 (18.6) | - 1.38 ^d^ |
| Family Cohesion (FC) | 34 | 76.18 (20.15) | 72.3 (21.6) | 0.18 |
| Physical Summary Measure (PhS) | 34 | 39.09 (18.39) | 53.0 (8.8) | - 1.41 ^d^ |
| Psychosocial Summary Measure (PsS) | 34 | 44.77 (9.87) | 51.2 (9.1) | - 0.70 ^e^ |
| **Children aged <5 years**  **ITQoL-SF47 subscale** |  |  |  |  |
| Parental Impact – Emotional (PE) | 37 | 68.41 (26.27) | 89.71 (16.47) | - 1.27 ^d^ |
| Parental Impact – Time (PT) | 37 | 79.95 (19.98) | 92.70 (17.59) | - 0.72 ^e^ |
|  |  |  |  |  |
| Physical Abilities (PA) | 37 | 87.75 (17.95) | 96.00 (15.99) | - 0.51 ^e^ |
| Growth and Development (GD) | 37 | 87.57 (20.09) | 94.68 (12.74) | - 0.55 ^e^ |
| Bodily Pain/Discomfort (BP) | 37 | 73.31 (23.78) | 83.07 (16.96) | - 0.57 ^e^ |
| Temperament and Moods (TM) | 37 | 79.37 (12.44) | 83.15 (12.39) | - 0.31 |
| Combined Behaviour (CBE)^c^ | 27 | 73.87 (15.41) | 77.49 (13.92) | - 0.26 |
| General Health Perceptions (GH)^f^ | 37 | 57.43 (18.36) | 78.17 (14.08) | -1.46 ^d^ |
| Family Cohesion (FC) | 36 | 80.97 (16.85) | 80.07 (20.98) | 0.04 |
| ^a^Effect size measures study means relative to population norms. Hedges’ *g* was calculated as follows: difference in group means (mean of the study sub-sample minus the ITQOL norm sample mean) divided by pooled standard deviation where pooled standard deviation = $\sqrt{\frac{\left( {SD}_{study sub-sample} \right)^{2}\left( n_{study sub-sample}-1 \right)+ \left( {SD}_{norm sample} \right)^{2}\left( n_{norm sample}-1 \right)}{\left( n_{study sub-sample}+ n_{norn sample} \right)-2}}$  ^b^ Higher scores represent higher HRQoL for all subscales.  ^c^ Scales/items answered only by parents of children aged 1 or older.  ^d^ Large, negative effect size (lower scores) relative to population norm. ‘Large’ effect size > -0.8  ^e^ Medium, negative effect size (lower scores) relative to population norm. ‘Medium’ effect size = 0.5 – 0.8.  ^f^ There were minor errors in the wording of questions contributing to this subscale. In question 8.1, a response option of “don’t know” was presented instead of “not sure”. In question 8.2, a response option of “much better than 1 year ago” was presented instead of “much better now than 1 year ago”.  ^^ Presented with results on caregiving-related QoL. | | | | |

**Supplementary Table 6.** Associations between child sex assigned at birth and child health-related quality of life

|  |  | **Child sex assigned at birth** | |  |
| --- | --- | --- | --- | --- |
|  |  | Female | Male |  |
|  | **N** | **Median (Q1-Q3)** | **Median (Q1-Q3)** | ***p*-value^a^** |
| **Children aged ≥5 years**  **CHQ-PF50 subscale**^b^ |  |  |  |  |
| Parental Impact – Emotional (PE) | 34 | 50.0 (25.0 - 58.3) | 37.5 (25.0 - 62.5) | 0.57 |
| Parental Impact – Time (PT) | 34 | 66.7 (55.6 - 88.9) | 66.7 (55.6 - 100) | 0.52 |
| Physical Functioning (PF) | 34 | 94.4 (27.8 - 100) | 91.7 (66.7 - 100) | 0.97 |
| Role/Social Limitations – Emotional/Behavoural (REB) | 34 | 88.9 (44.4 - 100) | 100 (61.1 - 100) | 0.34 |
| Role/Social Limitation – Physical (RP) | 34 | 100 (33.3 - 100) | 100 (50.0 - 100) | 0.83 |
| Bodily Pain/Discomfort (BP) | 34 | 75.0 (50.0 - 90.0) | 75.0 (60.0 - 80.0) | 0.45 |
| Behaviour (BE) | 34 | 73.8 (60.0 - 83.3) | 74.6 (62.1 - 82.1) | 0.77 |
| Mental Health (MH) | 34 | 67.5 (60.0 - 80.0) | 80.0 (67.5 - 85.0) | 0.10 |
| Self-Esteem (SE) | 34 | 70.8 (54.2 - 95.8) | 79.2 (70.8 - 89.6) | 0.54 |
| General Health Perceptions (GH) | 34 | 55.8 (35.0 - 66.7) | 48.8 (30.8 - 61.3) | 0.38 |
| Family Activities (FA) | 34 | 54.2 (37.5 - 83.3) | 72.9 (56.3 - 83.3) | 0.15 |
| Family Cohesion (FC) | 34 | 72.5 (60.0 - 85.0) | 85.0 (85.0 - 100) | 0.03 |
| Physical Summary Measure (PhS) | 34 | 47.0 (19.1 - 55.7) | 44.0 (29.8 - 51.9) | 0.63 |
| Psychosocial Summary Measure (PsS) | 34 | 44.6 (35.8 - 48.6) | 46.5 (39.6 - 54.8) | 0.39 |
| **Children aged <5 years**  **ITQoL-SF47 subscale** |  |  |  |  |
| Parental Impact – Emotional (PE) | 37 | 81.3 (62.5 - 87.5) | 75.0 (50.0 - 87.5) | 0.75 |
| Parental Impact – Time (PT) | 37 | 91.7 (75.0 - 100) | 83.3 (58.3 - 91.7) | 0.23 |
| Physical Abilities (PA) | 37 | 94.4 (88.9 - 100) | 93.3 (77.8 - 100) | 0.37 |
| Growth and Development (GD) | 37 | 97.5 (85.0 - 100) | 90.0 (85.0 - 100) | 0.59 |
| Bodily Pain/Discomfort (BP) | 37 | 75.0 (75.0 - 100) | 75.0 (62.5 - 75.0) | 0.11 |
| Temperament and Moods (TM) | 37 | 83.3 (75.0 - 91.7) | 79.2 (70.8 - 79.2) | 0.02 |
| Combined Behaviour (CBE)^c^ | 27 | 77.9 (68.8 - 86.3) | 77.9 (69.6 - 81.3) | 0.67 |
| General Health Perceptions (GH)^d^ | 37 | 60.0 (47.5 - 75.0) | 58.3 (47.5 - 64.2) | 0.38 |
| Family Cohesion (FC) | 36 | 85.0 (85.0 - 85.0) | 85.0 (85.0 - 85.0) | 0.39 |
| ^a^Calculated using the Wilcoxon rank-sum test  ^b^ Higher scores represent higher HRQoL for all subscales.  ^c^ Scales/items answered only by parents of children aged 1 or older.  ^d^ There were minor errors in the wording of questions contributing to this subscale. In question 8.1, a response option of “don’t know” was presented instead of “not sure”. In question 8.2, a response option of “much better than 1 year ago” was presented instead of “much better now than 1 year ago”. | | | | |
